# The relationship between non-high-density lipoprotein cholesterol to high-density lipoprotein cholesterol ratio (NHHR) and rheumatoid arthritis: a cross-sectional study

**DOI:** 10.3389/fnut.2025.1594218

**Published:** 2025-06-17

**Authors:** Shuai Deng, Yijian Qiu, Qiang Wang, Jingjing Jiang, Wang Xiang, Shifang Guo, Shanggeng Yang, Encheng Li, Qingsong Wu

**Affiliations:** ^1^Chengdu Sport University School of Sports Medicine and Health, Chengdu, China; ^2^Southwest Jiaotong University, College of Life Sciences and Engineering, Chengdu, China; ^3^Sichuan Integrative Medicine Hospital, Dermatology, Chengdu, China; ^4^Department of Orthopedics, Affiliated Sports Hospital of Chengdu Sport University, Chengdu, China

**Keywords:** NHHR, rheumatoid arthritis, NHANES, cross-sectional study, HDL-C, non-HDL-C

## Abstract

**Background:**

Research suggests a potential link between lipid metabolism and rheumatoid arthritis (RA). The ratio of non-high-density lipoprotein cholesterol (non-HDL-C) to high-density lipoprotein cholesterol (HDL-C) (NHHR) is a newly developed combined index to evaluate atherosclerotic lipids. This study aimed to explore the relationship between NHHR and the incidence rate of RA in the elderly in the United States.

**Methods:**

This study analyzed data from the National Health and Nutrition Examination Survey (NHANES) collected between 2005 and 2018 to examine the association between NHHR levels and RA incidence, adopting multivariate logistic, subgroup analysis, and smooth curve analysis methods. Furthermore, the study investigated the potential mediating role of BMI and the use of statins in the RA-NHHR association through mediation analysis. The nonlinear aspect of the RA-NHHR relationship was explored using restricted cubic spline (RCS) analysis.

**Results:**

This study enrolled 6,433 participants, with a mean age of 69.9 years. Males accounted for 51% of the cohort, while females comprised 49%. Among them, 1,359 individuals reported a history of RA. A one-unit increment in NHHR was associated with an 11% reduction in the risk of RA. [Odds ratio (OR): 0.87; 95% CI: 0.81–0.92]. We have extensively adjusted for various confounding factors and still reflect the above relationship. Subgroup analysis (all *p* for interaction < 0.05) showed that age, gender, race, smoking, drinking, statins, and diabetes had no significant impact on this negative correlation. Mediation analysis revealed a significant intermediary effect of NHHR on RA through statins (*p* for trend<0.01). Lastly, the RCS findings indicated a substantial non-linear association between NHHR and RA (*p*-non-linear<0.05). Due to the cross-sectional nature of this study, it is not possible to determine the causal relationship. Therefore, in future studies, we can utilize other research designs, such as prospective cohort studies, to validate the temporal relationship between the two.

**Conclusion:**

Research has shown that NHHR levels are associated with a decrease in the incidence of rheumatoid arthritis in the elderly population in the United States. In addition, the linear relationship between RA and NHHR warrants further study.

## 1 Introduction

Rheumatoid arthritis (RA) is a long-lasting autoimmune disease that causes joint inflammation. It mainly affects multiple joints on both sides of the body, leading to pain, morning stiffness, and swelling (1). The persistent inflammatory microenvironment accelerates cartilage erosion and bone resorption, ultimately leading to irreversible joint deformity in approximately 30 percent of patients within 2 years of onset (2). Elderly-onset rheumatoid arthritis (EORA) occurs in people over 60 years old and shows different symptoms compared to younger patients. This age group tends to have more severe reactions, with a 1.5 to 2-fold increase in C-reactive protein (CRP) levels. They also have a higher chance of experiencing symptoms outside the joints, with about twice the likelihood compared to younger people. Additionally, older patients respond less effectively to standard treatments for RA, with a 17 to 23 percent lower response rate when using traditional disease-modifying antirheumatic drugs (DMARDs) (3, 4). This age-related heterogeneity may be due to the cumulative effects of immunosenescence and inflamm-aging due to thymic degeneration (5).

Epidemiological studies show that the prevalence of rheumatoid arthritis (RA) is between 0.5 and 1% in the general population and rises to 2.3% in those aged 65 and older (6). The risk of cardiovascular events in older individuals with RA is 1.5 to 2.0 times higher than in similar-age controls, primarily due to atherosclerosis (7). This phenomenon may be related to the accelerated vascular endothelial dysfunction of chronic inflammation associated with RA, in which lipid metabolism disorders play a key role (8). Recent research shows that the non-HDL cholesterol to HDL cholesterol ratio (NHHR) better indicates atherosclerotic lipid profiles than traditional measures. Non-HDL-C includes all atherogenic lipoproteins, while HDL-C is anti-inflammatory and vasoprotective through reverse cholesterol transport (9, 10).

Despite the available evidence, characteristic changes such as decreased HDL-C levels (15 to 20 percent) and increased oxidatively modified lipoproteins are common in patients with RA (11). However, there is still a gap in knowledge about the association between NHHR and RA. Of particular concern is the fact that the pathological significance of NHHR may be further amplified by age-related declines in lipoprotein-metabolising enzyme activity (eg, a 30 to 40 percent decrease in liver esterase activity) and changes in lipid profile due to estrogen deficiency in older adults (12). Based on the above background, we assume that there is a specific relationship between NHHR and RA this study used the data of the National Health and Nutrition Survey (NHANES) from 2005 to 2018 for the first time, aiming to explore the dose–response relationship between NHHR and RA risk in the elderly population (≥ 60 years old), and to provide an epidemiological basis for the establishment of an early warning system for RA based on the regulation of lipid metabolism.

## 2 Methods

### 2.1 Study population

In this study, we used NHANES data from 2005 to 2018. NHANES is a national health and nutrition survey aimed at measuring the health and nutrition status of adults and children in the United States. It is the only national health survey that includes health checks and laboratory tests for participants of all age groups (13). Survey data drives changes in how doctors treat patients and how public policies support good health. These data can be publicly accessed through the NHANES website.

**Figure 1:**
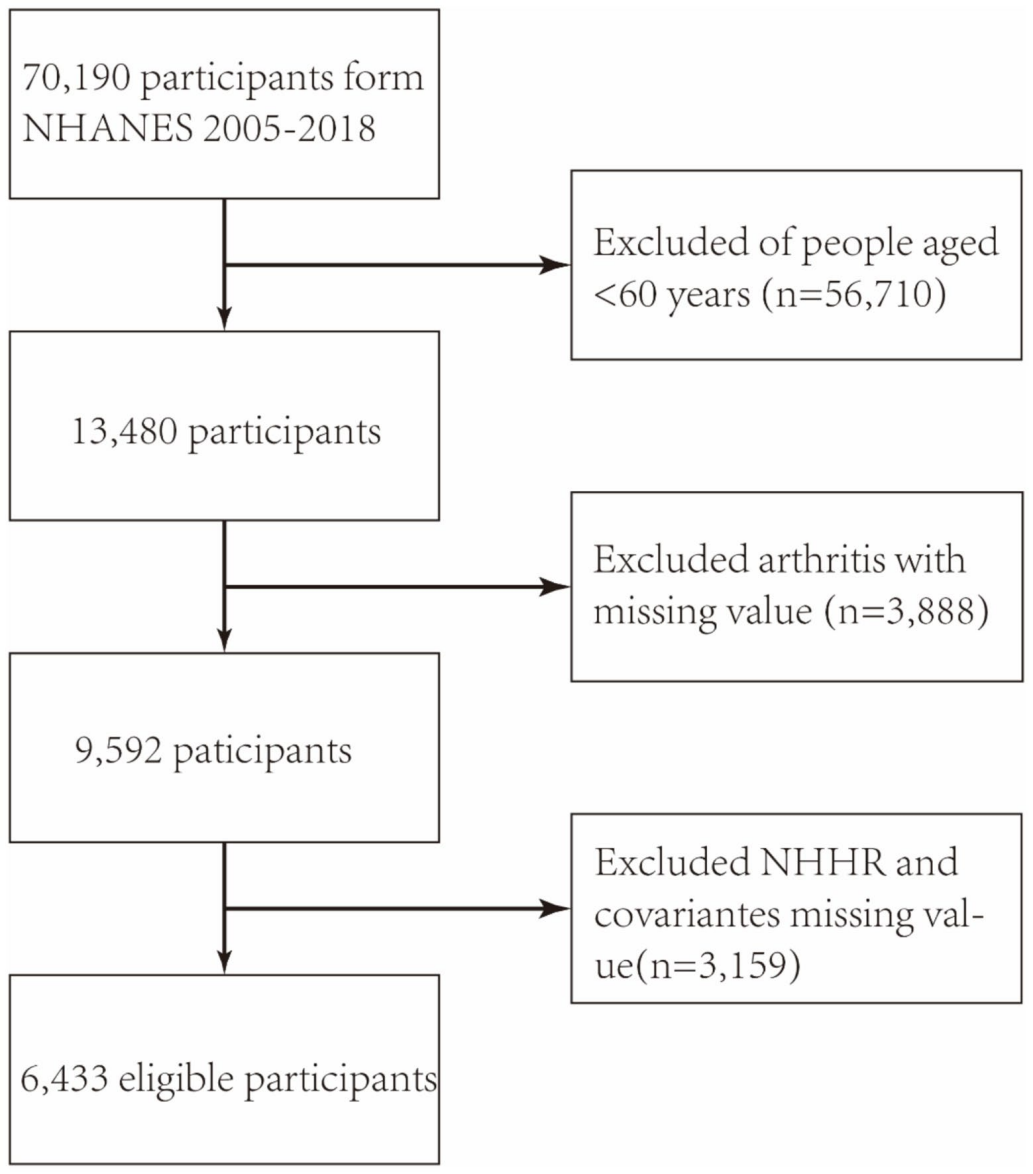
The flowchart depicts the participant selection process in the NHANES study conducted from 2005 to 2018. NHHR: non-high-density lipoprotein cholesterol-to-high-density lipoprotein cholesterol ratio.

As shown in Figure 1, the study used NHANES data from 2005 to 2018, initially including 70,190 participants. The exclusion was based on specific demographic characteristics: (a) patients under 60 years old (*n* = 56,710), (b) patients with missing arthritis data (*n* = 3,888), and (c) patients without NHHR data (*n* = 3,153). Finally, 6,433 individuals were included in the analysis.

### 2.2 Exposure factors

NHHR is included as an independent variable. The calculation of NHHR comes from laboratory data named “HDL. Doc” and “TCHOL. Doc” in NHANES. Subtract HDL from the dataset’s total cholesterol, then divide the difference between the two by HDL (14).

### 2.3 Definition of ending

RA is the outcome indicator of this study, and the diagnosis of arthritis is obtained through questionnaire data (MCQ16a). Specifically, participants were asked, “Has a doctor or other health professional ever told you that you had arthritis?” and the answer options were “Yes” or “No.” Next, diagnose whether the participant has RA through the following questions. “Which type of arthritis was it?”。 The answer options are “rheumatoid arthritis,” “osteoarthritis,” “psoriatic arthritis,” “other,” “refuse,” and “do not know.” We will designate participants who answer “Yes” to the first question and “Rheumatoid Arthritis” to the second question as the disease group and participants who answer “No” to the first question as the control group. A previous study showed an 85% consistency between self-reported and clinically confirmed arthritis (15).

### 2.4 Covariates

To investigate the association between NHHR and rheumatoid arthritis (RA), potential confounders were addressed by adjusting for gender, age, race, educational attainment, smoking status, alcohol consumption, poverty income ratio (PIR), body mass index (BMI), and diabetes. Smoking status is determined based on the NHANES questionnaire survey (SMQ040), where participants’ responses of “Every day” or “Some days” are defined as smoking, while other responses are defined as non-smoking. Alcohol use is evaluated based on “Ever have 4/5 or more drinks every day. The status of diabetes is determined based on the answers to the NHANES questionnaire. Individuals who meet any of the following criteria are considered to have diabetes: they are told by the doctor that they have diabetes, are currently taking anti-diabetes drugs, and have glycosylated hemoglobin levels exceeding 6.5% or fasting glucose levels above 126 mg/dL. At the same time, we collected medication data from participants through the NHANES database, categorizing those who have taken statins as using medication, and those who have used other medications or do not use medication as not using statins. The education level is divided into “Below High School” and “Below High School.” The BMI categories are <25 kg/m^2^, 25–30 kg/m^2^, and ≥ 30 kg/m^2^. The three PIR levels are “<1.3”, “1.3–3.5”, and “≥ 3.5.”

### 2.5 Statistical analysis

We apply appropriate statistical weights to adjust the NHANES design. The statistical analysis in this study was conducted using Empower software^1^ and R software (version 4.4.2). Continuous data are described as mean ± standard deviation (SD), while categorical data are reported as proportions (%). NHHR was categorized into quartiles, using the lowest quartile (Q1) as the reference. A multivariate logistic regression model was employed to assess the association between NHHR and RA, incorporating both continuous and quartile measures of NHHR as independent variables. This study used three models to investigate the independent relationship between NHHR and RA. Model 1 was unadjusted. Model 2 was adjusted for gender, age, and race, while Model 3 additionally accounted for educational attainment, PIR, BMI, alcohol consumption, smoking status, statins, and diabetes. Then, subgroup analysis will investigate gender, age, race, PIR, MBI, potential educational differences, and other factors. And through mediation analysis, to examine whether the impact of NHHR on RA is transmitted through BMI. Reveal the causal mechanisms between variables.

## 3 Results

### 3.1 Baseline characteristics of participants

Table 1 summarizes the demographic and clinical characteristics of NHANES participants (2005–2018) stratified by NHHR quartiles. The definition of quartiles is as follows: Q1 (<1.92), Q2 (1.92–2.68), Q3 (2.68–3.66), Q4 (>3.66). This study included 6,433 participants with an average age of 69.9 years, of which 51% were male and 49% were female. 1,359 participants reported a history of RA. Participants with higher NHHR values are more likely to be male, non-Hispanic White, have no smoking history, have received a high school education or higher, have not used statins, and have a BMI of 25–30 (all *p* < 0.05). According to age, gender, race, drinking, smoking, education, diabetes, BMI, RA, and NHHR, there were significant differences between the quartiles (*p* < 0.01).

**Table 1:** Baseline demographic and clinical features of NHANES participants (2005–2018).

Characteristic	Total	Q1 (<1.92)	Q2 (1.92, 2.68)	Q3 (2.68, 3.66)	Q4 (>3.66)	*p*-value
*N*	6,433	1,604	1,602	1,603	1,624	
Age	69.9 ± 7.2	70.53 ± 7.12	70.04 ± 7.03	68.97 ± 6.92	68.26 ± 6.71	<0.001
Gender						<0.001
Male	2,942(51)	755 (39.51)	820 (46.26)	904 (52.78)	1,012 (58.08)	
Female	3,491 (49)	849 (60.49)	782 (53.74)	699 (47.22)	612 (41.92)	
Race						<0.001
Mexican American	794 (4.15)	128 (2.89)	173 (3.71)	217 (4.37)	276 (5.74)	
Other Hispanic	550 (3.35)	93 (2.24)	122 (3.02)	141 (3.53)	194 (4.69)	
Non-Hispanic White	3,223 (78.38)	825 (79.1)	828 (78.59)	795 (79.22)	775 (76.53)	
Non-Hispanic Black	1,302 (8.14)	407 (10.08)	346 (8.92)	303 (7.35)	246 (6.08)	
Other Race	564 (5.98)	151 (5.7)	133 (5.77)	147 (5.54)	133 (6.96)	
Alcohol						0.025
Yes	863 (12.42)	190 (10.17)	206 (11.96)	217 (12.86)	250 (14.85)	
No	5,570 (78.58)	1,414 (89.83)	1,396 (88.04)	1,386 (87.14)	1,374 (85.15)	
Smoke						<0.001
Yes	835 (11.45)	206 (10.57)	180 (10.11)	188 (10.74)	261 (14.5)	
No	5,598 (88.55)	1,398 (89.43)	1,422 (89.89)	1,415 (89.26)	1,363 (85.5)	
Education						0.0003
Below high school	1941 (19.19)	425 (16.42)	462 (19.31)	487 (18.85)	567 (22.37)	
High School or above	4,492 (80.81)	1,179 (83.58)	1,140 (80.69)	1,116 (81.15)	1,057 (77.63)	
Diabetes						0.268
Yes	1877 (23.7)	463 (23.13)	444 (21.94)	469 (21.74)	501 (28.13)	
No	4,556 (76.3)	1,141 (76.87)	1,158 (78.06)	1,134 (78.26)	1,123 (71.87)	
Statins						<0.001
Yes	3,967 (39.75)	827 (53.41)	703 (44.69)	575 (36.3)	361 (23.63)	
No	2,466 (60.25)	777 (46.59)	899 (55.31)	1,028 (63.7)	1,263 (76.37)	
BMI						<0.001
<25	1915 (29.83)	720 (47.02)	496 (30.9)	410 (23.37)	289 (17.01)	
> = 25, <30	2,436 (37.63)	538 (32.3)	602 (37.93)	603 (40.13)	693 (40.44)	
> = 30	2082 (32.54)	346 (20.68)	504 (31.17)	590 (36.5)	642 (42.55)	
PIR						<0.001
<1.3	2,200 (23.32)	494 (21.32)	531 (22.8)	577 (25.2)	598 (24.06)	
> = 1.3, <3.5	2,452 (37.8)	609 (38.14)	622 (38.42)	592 (35.67)	629 (39.01)	
> = 3.5	1781 (38.88)	501 (40.54)	449 (38.78)	434 (39.13)	397 (36.94)	
RA						<0.001
No	5,074 (78.51)	1,221 (77)	1,256 (78.47)	1,251 (77.97)	1,346 (80.74)	
Yes	1,359 (21.49)	383 (23)	346 (21.53)	352 (22.03)	278 (19.26)	

NHHR, non-high-density lipoprotein cholesterol to high-density lipoprotein cholesterol ratio; BMI, body mass index; PIR, poverty-income ratio; RA, rheumatoid arthritis.

### 3.2 The association between the NHHR and RA

Table 2 highlights the association between NHHR and rheumatoid arthritis (RA), revealing an inverse correlation between higher NHHR levels and lower RA prevalence in the continuous mode. Specifically, in Model 1 (the unadjusted model), a one-unit increase in NHHR was associated with an 11% decrease in RA prevalence [odds ratio (OR): 0.89; 95% confidence interval (CI): 0.85–0.94]. This inverse relationship persisted in Model 2 (partially adjusted model) and Model 3 (fully adjusted model) (Model 2: OR: 0.95; 95% CI: 0.90–1.00; Model 3: OR: 0.92; 95% CI: 0.88–0.99). In the categorical analysis, compared to the lowest NHHR quartile (Q1), the highest quartile (Q4) exhibited a 34% reduction in RA prevalence in Model 1 (OR: 0.66; 95% CI: 0.55–0.78). Similarly, in Model 2, Q4 showed an 18% decrease in RA prevalence compared to Q1 (OR: 0.82; 95% CI: 0.69–0.98). In Model 3, Q4 demonstrated a 28% reduction in RA prevalence relative to Q1 (OR: 0.78; 95% CI: 0.64–0.94). These findings suggest a consistent and statistically significant inverse relationship between NHHR levels and RA risk across multiple analytical models.

**Table 2:** Weighted logistic regression analysis of the NHHR-RA association.

Exposure	OR (95%CI)		
	Model 1 (*n* = 6,433)	Model 2 (*n* = 6,433)	Model 3 (*N* = 6,433)
NHHR	0.89 (0.85, 0.94) < 0.0001	0.95 (0.90, 1.00) 0.0410	0.93 (0.88, 0.99) 0.0145
Quartile			
Quartile 1	Reference	Reference	Reference
Quartile 2	0.88 (0.74, 1.04) 0.1237	0.93 (0.78, 1.10) 0.3957	0.89 (0.75, 1.06) 0.1876
Quartile 3	0.89 (0.76, 1.06) 0.1962	1.04 (0.87, 1.23) 0.7689	0.97 (0.82, 1.16) 0.765
Quartile 4	0.66 (0.55, 0.78) < 0.0001	0.82 (0.69, 0.98) 0.0305	0.78 (0.64, 0.94) 0.0098
P for trend	<0.0001	0.0587	0.0207

Model 1: Not adjusted. Model 2: Adjust based on gender, age, and race. Model 3: Based on gender, age, race, education level, smoking, alcohol consumption, statins, PIR, BMI, and diabetes. Hierarchical analysis not adjusted for gender.

### 3.3 Smoothed curve fitting analysis

**Figure 2:**
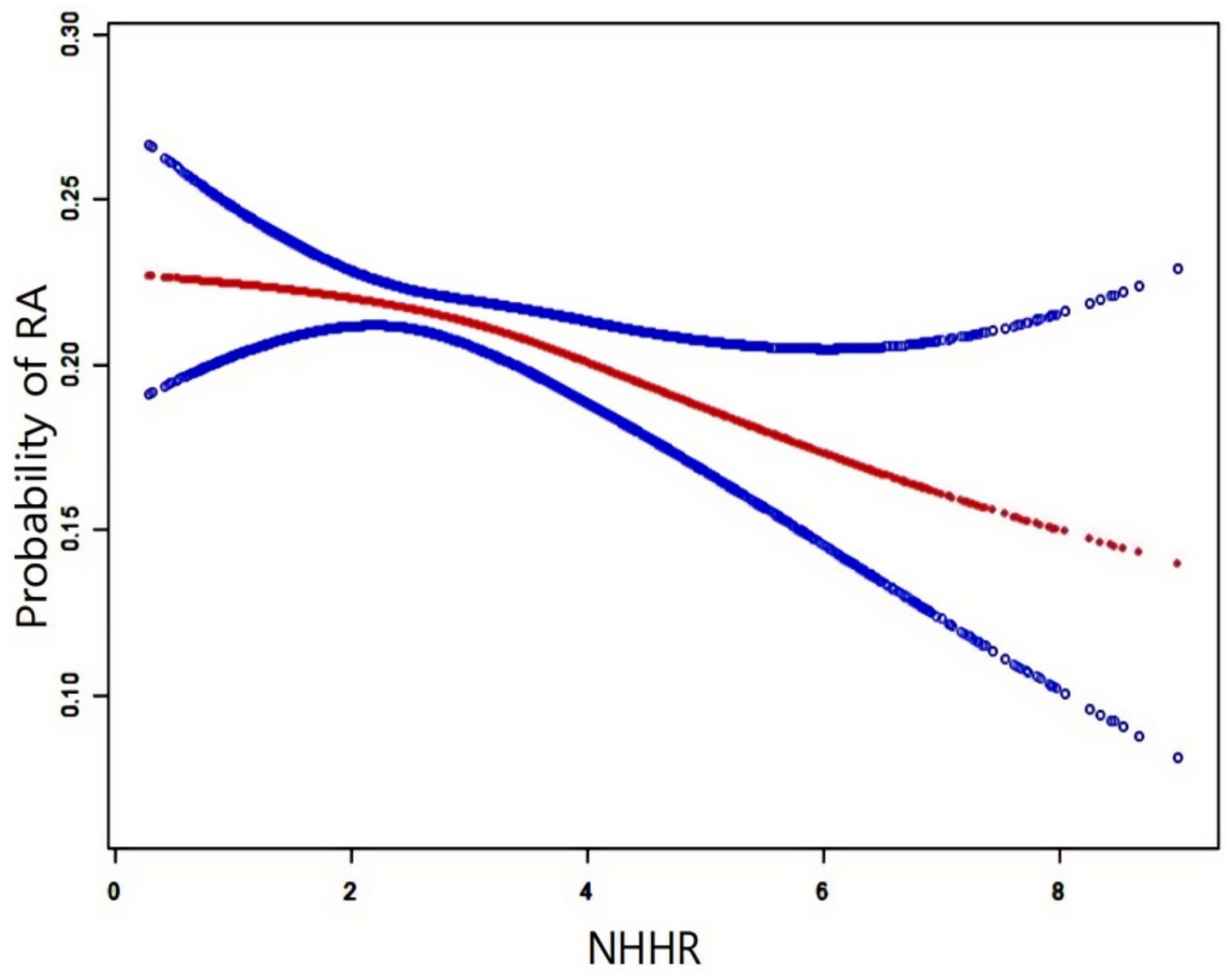
Smooth curve between NHHR and RA.

Figure 2 illustrates the NHHR-RA probability relationship analyzed using smooth curve fitting, with adjustments for age, gender, race, diabetes, educational attainment, smoking status, PIR, alcohol consumption, statins, and BMI. The results confirmed a negative correlation between NHHR and RA development.

The red solid line indicates the smooth curve fit, while the blue band depicts the 95% confidence interval. The model accounted for gender, age, race, educational attainment, smoking status, alcohol use, hypertension, statins, PIR, and BMI.

### 3.4 Subgroup analyses

**Figure 3:**
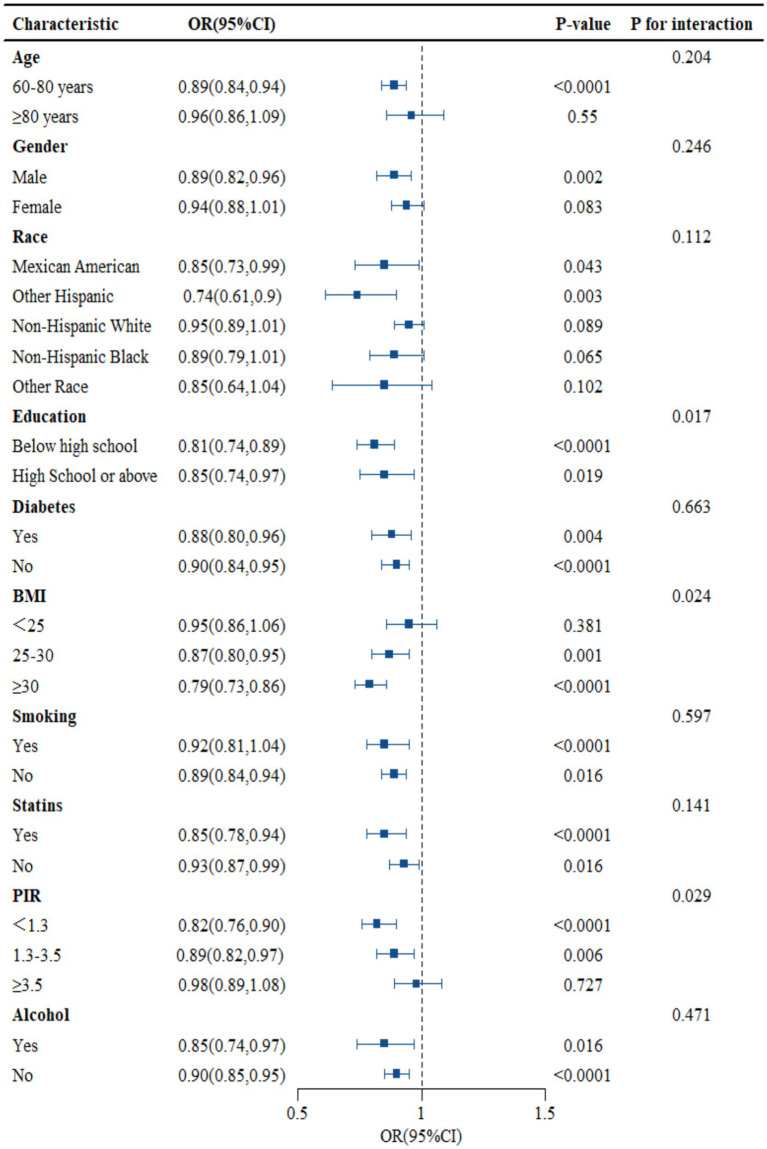
Subgroup analysis of the association between NHHR and RA.

As shown in Figure 3, subgroup analysis results showed that education, PIR, and BMI changed the association between NHHR and RA (*p* < 0.05), but age, gender, race, smoking, drinking, statins, and diabetes did not alter the association between the two (*p* > 0.05). The data in Figure 3 shows that, except for education, PIR, and BMI, the probability of NHHR growth accompanied by RA decline may be consistent across subgroups (see Figure 3).

### 3.5 Mediation analysis

The mediation analysis results show that NHHR has a significant adverse total effect on RA [−0.027 (*p* < 0.05)], and ADE [−0.0466 (*p* < 0.05)] indicates that NHHR hurts RA. After controlling for statins, the ACME of NHHR on RA [−0.006 (*p* < 0.05)] remains negative. The mediation ratio indicates that NHHR not only has a direct negative impact on RA but also exacerbates the negative impact on RA through mediating factors (statins) (Table 3).

**Table 3:** Using statin medication as a mediator variable.

Effect type	Adjusted model	
	Estimate (95% CI)	*p*-value
Total effect	−0.015	<0.05
ACME	−0.003	<0.05
ADE	−0.012	<0.05

ACME, Averaged Casual Mediation Effects; ADE, Average Direct Effect; Prop. Mediated, Proportion Mediated. Model adjusted for age, gender, race, education, diabetes, alcohol, PIR, and BMI.

### 3.6 Restricted cubic spline (RCS)

Relationships in the logistic regression model were assessed with restricted cubic spline (RCS) strips located at the five nodes of the exposure distribution at the 20, 40, 60, 80, and 100 percentiles, and the median VAI was used as the reference value for odds ratio (OR) = 1.

**Figure 4:**
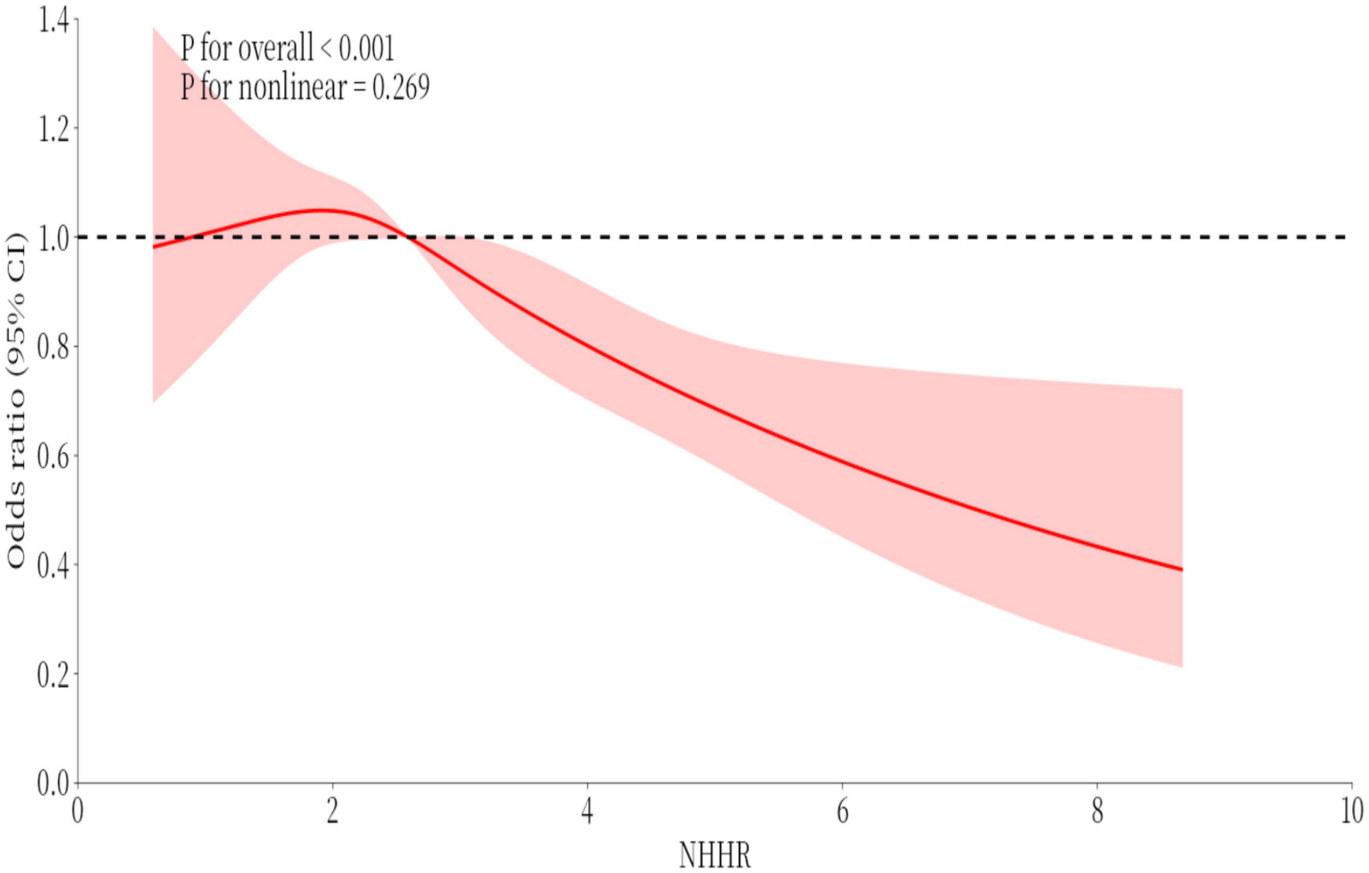
Restricted cubic spline (RCS).

To investigate potential nonlinear relationships between RA and NHHR prevalence, restricted cubic spline (RCS) analysis was employed (Figure 4). The results indicated an insignificant nonlinear relationship between RA and NHHR (p-non-linear = 0.269).

## 4 Discussion

Our study provides novel insights into the inverse association between NHHR and rheumatoid arthritis (RA) prevalence in older U. S. adults (≥60 years), highlighting the critical role of lipid metabolism in age-related autoimmune pathogenesis. Notably, each unit increase in NHHR was associated with an 11% reduction in RA risk, a relationship that persisted across all subgroups and remained robust after adjusting for confounders. These findings not only expand the clinical utility of NHHR beyond cardiovascular risk assessment but also underscore its potential as a biomarker for RA susceptibility in aging populations.

The focus on individuals aged ≥60 years is of particular clinical relevance. Aging is characterized by immunosenescence and inflamm-aging (16), processes marked by chronic low-grade inflammation and dysregulated immune responses, which amplify RA severity and accelerate joint destruction in elderly-onset RA (EORA) patients (17). Compared to younger cohorts, EORA patients exhibit higher CRP levels (1.5–2-fold elevation), increased prevalence of extra-articular manifestations (18), and reduced responsiveness to conventional DMARDs (17–23% lower efficacy) (19). These age-related disparities may stem from thymic involution, altered T-cell repertoire, and cumulative oxidative stress, all exacerbating lipid metabolism disturbances (20). Importantly, elderly individuals experience age-dependent declines in hepatic lipase activity (30–40% reduction) and estrogen deficiency-induced shifts in lipoprotein composition (21), rendering NHHR a more sensitive indicator of inflammatory-metabolic crosstalk in this population. Our stratified analysis further confirmed the consistency of the NHHR-RA association across age subgroups, reinforcing its clinical applicability in geriatric care.

While reduced HDL-C and elevated oxidized LDL are well-documented in RA (22), NHHR integrates both atherogenic (non-HDL-C) and atheroprotective (HDL-C) components, offering a dynamic metric to evaluate systemic inflammation’s impact on lipid homeostasis (23). Mechanistically, pro-inflammatory cytokines (e.g., IL-6, TNF-*α*) may drive this association by enhancing hepatic LDL receptor expression and HDL dysfunction, lowering circulating atherogenic particles while diminishing anti-inflammatory HDL functionality (24). Research has compellingly demonstrated that individuals experiencing disease progression have significantly elevated levels of apolipoprotein B (Apo B) lipoprotein subclasses that include apolipoprotein C-III (Apo C-III) when compared to non-progressors. Moreover, numerous studies have established a strong association between these identical lipoproteins and an increased coronary artery calcification score (CAC) in progressive patients. This evidence positions these lipoproteins as critical new risk factors for the advancement of atherosclerosis in patients with RA, underscoring the urgent need for further investigation and targeted clinical strategies (25, 26). Clinically, NHHR’s predictive value extends beyond RA risk stratification: its integration into routine screening could enable early identification of high-risk elderly individuals, particularly those with subclinical inflammation or metabolic comorbidities. Furthermore, the mediation effect of statins suggests that statins may synergize with lipid-modifying therapies to mitigate RA progression in older adults.

As a cross-sectional study, causal relationships cannot be inferred; A prospective cohort is needed to verify the temporal relationship between NHHR and the onset of RA. Unmeasured confounding factors, such as genetic factors and microbiome interactions, limit the explanation of the mechanism. Since the NHANES database does not include data on inflammatory markers such as CRP and RF, the association between the NHHR-RA two may be affected. Future research can explore the molecular interactions between NHHR and RA-specific autoantibodies (such as anti-CCP, RF), and validate their predictive performance in different ethnic cohorts.

## 5 Conclusion

This study establishes NHHR as a promising biomarker for RA susceptibility in older adults, bridging the gap between lipid dysregulation and age-exacerbated autoimmunity. By prioritizing NHHR monitoring in geriatric populations, clinicians may enhance early detection and implement personalized preventive strategies, ultimately reducing the dual burden of RA and cardiovascular morbidity in aging societies.

## Data Availability

The datasets presented in this study can be found in online repositories. The names of the repository/repositories and accession number(s) can be found below: https://www.cdc.gov/nchs/nhanes.

## Footnotes

^1^ http://www.empowerstats.com
